# Association of Sustained Response Duration With Survival After Conventional Transarterial Chemoembolization in Patients With Hepatocellular Carcinoma

**DOI:** 10.1001/jamanetworkopen.2018.3213

**Published:** 2018-10-05

**Authors:** Yaojun Zhang, Mengping Zhang, Minshan Chen, Jie Mei, Li Xu, Rongping Guo, Xiaojun Lin, Jiaping Li, Zhenwei Peng

**Affiliations:** 1 Cancer Center, Sun Yat-sen University, Guangzhou, China; 2 State Key Laboratory of Oncology in South China, Guangzhou, China; 3 Collaborative Innovation Center for Cancer Medicine, Guangzhou, China; 4 Department of Oncology, The First Affiliated Hospital of Sun Yat-sen University, Guangzhou, China; 5 Clinical Trials Unit, The First Affiliated Hospital of Sun Yat-sen University, Guangzhou, China; 6 Department of Interventional Oncology, The First Affiliated Hospital of Sun Yat-sen University, Guangzhou, China

## Abstract

**Importance:**

Defining early reliable surrogate end points for survival in patients with hepatocellular carcinoma (HCC) after conventional transarterial chemoembolization (cTACE) is of great value.

**Objective:**

To evaluate the association between sustained response duration (SRD) and overall survival (OS) after cTACE for intermediate HCC.

**Design, Setting, and Participants:**

This multicenter cohort study enrolled 2403 consecutive patients in China with naive intermediate HCC between June 1, 2000, and December 31, 2008, as the primary cohort, and 331 consecutive patients with intermediate naive HCC between January 1, 2011, to June 30, 2012, as the validation cohort. All patients received cTACE as an initial treatment. Initial response and best response were defined as the radiological response after first cTACE or best radiological response after 2 or more sessions of cTACE, respectively. Responders were those who experienced complete response or partial response. Sustained response duration was defined as the time between the date when complete response, partial response, or stable disease was achieved and the date progressive disease occurred after cTACE. Response was evaluated by modified Response Evaluation Criteria in Solid Tumors. Information about patients in the study was collected from January 1, 2018, to March 31, 2018, and analysis of these data was performed in April 2018.

**Main Outcomes and Measures:**

Overall survival.

**Results:**

A total of 2734 total patients (2499 of 2734 [91.4%] male; median [range] age, 56.5 [18-75] years) were included in the analysis. In the primary cohort, SRD of 6 months or more was found to have the strongest association with 5-year OS after cTACE among different durations of sustained response. Patients with SRD of 6 months or more (387 of 430 male; median [range] age, 57 [18-75] years) had the longest median (range) OS (67.7 [64.8-72.1] months), followed by initial responders (760 of 874 male; median [range] age, 56 [18-75] years; median [range] OS, 55.8 [55.0-57.7] months) and best responders (939 of 1032 male; median [range] age, 57 [18-75] years; median [range] OS, 53.2 [52.2-54.6] months). Response duration of 6 months or more was found to be an independent prognostic factor for OS (hazard ratio, 0.145; 95% CI, 0.124-0.170; *P* < .001). The significance of SRD as a factor associated with OS was confirmed in the validation cohort.

**Conclusions and Relevance:**

Sustained response duration of 6 months or more was associated with OS and may serve as an early surrogate end point after cTACE for intermediate HCC.

## Key Points

**Question:**

Is there an early reliable surrogate end point for overall survival (OS) in patients with hepatocellular carcinoma after conventional transarterial chemoembolization?

**Findings:**

In this cohort study of 2734 patients in China, sustained response duration of 6 months or more was found to have the strongest association with 5-year OS after chemoembolization. Sustained response duration of 6 months or more was found to be the independent prognostic factor for OS.

**Meaning:**

Sustained response duration of 6 months or more was associated with OS and may serve as an early surrogate end point after conventional transarterial chemoembolization for intermediate hepatocellular carcinoma.

## Teaser

This cohort study of patients in China investigates whether sustained response duration of 6 months or more following treatment with conventional transarterial chemoembolization (cTACE) is a reliable surrogate end point for overall survival in patients with intermediate hepatocellular carcinoma (HCC).

## Introduction

Hepatocellular carcinoma (HCC) is a global, continuously growing public health problem.^1,2^ Conventional transarterial chemoembolization (cTACE) has been widely recognized as the mainstay of therapy for intermediate HCC according to the Barcelona Clinic Liver Cancer staging system.^1,2,3,4,5^

According to modified Response Evaluation Criteria in Solid Tumors (mRECIST), radiological response is the most common criterion used to evaluate efficacy of cTACE on the basis of tumor viability.^6^ Generally, necrosis response is postulated to be an early surrogate for therapeutic benefit after treatment.^7,8^ According to mRECIST, complete response (CR) after initial cTACE is considered to be a predictor for favorable outcome.^9^ However, radiological CR after cTACE does not always match histological complete tumor necrosis.^10^ Existence of residual viable tumor cells that are just stunned and can later recover, causing relapse, has been hypothesized.^7,8^ In addition, the embolic nature of cTACE causing hypoxia can result in upregulation of vascular endothelial growth factor.^11^ Upregulation of vascular endothelial growth factor or cytokines can increase tumor angiogenesis or tumor cell proliferation, contributing to treatment failure.^7^ These facts imply that mRECIST might exaggerate or overestimate the efficacy of cTACE based on early image response. Furthermore, what should be done if a patient does achieve CR or partial response (PR) after first cTACE according to mRECIST? Two treatment strategies have been proposed: treatment on schedule or treatment on demand. There is a controversy and a lack of evidence to suggest which approach is superior.^8,12^ If an early response is able to predict survival in HCC, adjustments in patient management can be made early in the follow-up period to achieve the best balance between tumor control and quality of life. Therefore, defining reliable surrogate end points for survival in patients with HCC after cTACE is of great value. Sustained CR has been demonstrated to be associated with favorable outcome after local therapy, such as radiofrequency ablation.^13^ This implies that maintaining the status of response, rather than achieving the robust response itself, may be more clinically important for survival after cTACE. To date, we know of no studies exploring the role of sustained response duration (SRD), defined as the time between the date when CR, PR, or stable disease is achieved and the date progressive disease occurs in predicting clinical prognosis of HCC after cTACE. Therefore, we conducted this large cohort study to evaluate the prognostic role of SRD in HCC after cTACE and validated its value among a cohort of patients with HCC from another tertiary medical center in China.

## Methods

### Patients

**Figure 1.**
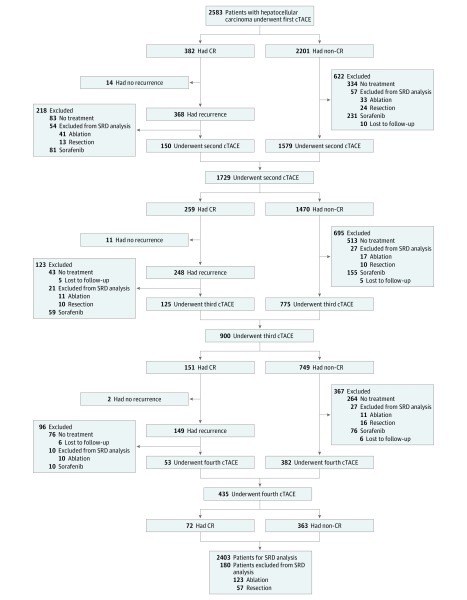
Repetition of Conventional Transarterial Chemoembolization (cTACE) in the Primary Cohort Patients received repeated cTACE until 1 of the following occurred: (1) complete response (CR); (2) technical impossibility to embolize the residual tumor, for example, in a tumor only supplied by extrahepatic collateral arteries; (3) development of contraindications to cTACE; or (4) total resection or ablation of tumor by subsequent surgery or local ablation. If progressive disease occurred, sorafenib was recommended. SRD indicates sustained response duration.

This is a retrospective cohort study on prospectively collected data in 2 tertiary medical centers. The primary cohort included 2583 consecutive patients with intermediate naive HCC who underwent cTACE at the Cancer Center of Sun Yat-sen University between June 1, 2000, and December 31, 2008. From January 1, 2011, to June 30, 2012, 394 consecutive patients with intermediate naive HCC who received cTACE in the First Affiliated Hospital, Sun Yet-sen University, were enrolled as the validation cohort. Diagnosis of HCC was based on histological test results or the noninvasive criteria used by the American Association for the Study of Liver Diseases.^2^ The inclusion criteria were (1) age between 18 and 75 years; (2) Child-Pugh grades A5, A6, or B7; (3) Eastern Cooperative Oncology Group performance score 0; and (4) HCC with no previous treatment. The exclusion criteria were (1) underwent curative therapies (liver transplantation, resection, or ablation) during follow-up; (2) Child-Pugh grades higher than B7; and (3) patients missing radiological images (Figure 1). This exclusion was performed to create a homogeneous cohort with few confounding variables and minimize the number of patients with negative tumor biology. This study was approved by the institutional ethics committees of both centers. Informed consent was waived due to the retrospective nature of the study. We reported this study according to the Strengthening the Reporting of Observational Studies in Epidemiology (STROBE) reporting guideline.

### Conventional Transarterial Chemoembolization 

According to previous studies,^14,15,16^ cTACE was performed within 1 to 3 days after diagnosis of HCC. Hepatic artery infusion chemotherapy was performed using carboplatin, 150 to 300 mg. Then chemolipiodolization was performed as selectively as possible, treating individual segmental arteries when feasible. When more than 1 target branch was present, such as bilobar tumor, chemolipiodolization was divided proportionately, depending on the size and number of vessels to be treated, and administered sequentially into the target vessels, which were then recatheterized and embolized with absorbable gelatin sponge particles 1 to 2 mm in diameter to achieve stasis. Stasis was defined as the absence of antegrade flow within a vessel such that contrast filling the target vessel persisted, without washout, 5 cardiac beats after the injection of contrast.^17,18^ Chemolipiodolization was performed using epirubicin, 30 to 50 mg, and mitomycin C, 6 to 8 mg, mixed with 5 to 15 mL of lipiodol. The doses of chemotherapy drugs were dependent on each patient’s renal function, total bilirubin, and indocyanine green retention at 15 minutes.

### Response Assessment

Radiological assessment of response was performed 4 weeks after cTACE using cross-sectional contrast-enhanced computed tomography (CT) or gadolinium-enhanced magnetic resonance imaging (MRI), and then once every 2 to 3 months. For each patient, the imaging modality (MRI vs CT) remained the same throughout the study period. The radiological results were analyzed by 2 independent radiologists in each center, and both of them were blinded to patients’ clinical data and clinical outcomes.

Treatment response was assessed using mRECIST guidelines.^6^ *Responders* referred to objective response, namely the sum of patients who experienced CR or PR. *Nonresponders* referred to the sum of patients who had stable disease (SD) and progressive disease (PD). *Initial response* was defined as the radiological response after first cTACE. *Best response* was defined as best radiological response across repeated cTACE sessions. Patients who achieved initial or best response were considered as initial or best responders, respectively. Sustained response duration, defined in the Introduction, is illustrated by the following example: patient reached SD after first session of cTACE, PR after the second cTACE session 3 months later, CR after the third cTACE session 3 months after the second, and PD after the last session another 3 months later. The SRD for this patient was the time between achieving SD and reaching PD (9 months). Patients developing new lesions, vascular invasion, and/or metastases were categorized as having PD. Patients were divided into 4 groups based on SRD: SRD of 12 months or longer, SRD between 6 and 12 months, SRD between 3 and 6 months, and SRD of less than 3 months.

### Treatment Strategy After First Session of cTACE

During follow-up, repeated cTACE treatments were performed on demand upon the demonstration of a viable tumor (non-CR) or local and/or distant intrahepatic recurrences in patients with Child-Pugh scores of A5, A6, and B7 who were clinically suitable for cTACE treatment until 1 of the following end points was reached: (1) CR; (2) technical impossibility to embolize the residual tumor, for example, in a tumor only supplied by extrahepatic collateral arteries; (3) development of contraindications to cTACE; or (4) total resection or ablation of tumor by subsequent surgery or local ablation. In the second and third scenarios, patients were recommended to receive sorafenib. If they refused, the best supportive treatment was given. For patients with PD after the first cTACE, repeat cTACE was performed. If PD occurred after the second cTACE, sorafenib was recommended. If patients refused, the best supportive treatment was provided.

### Statistical Analysis

Overall survival (OS) was estimated from the date of initial diagnosis of HCC to the date of death from any cause or the last follow-up. The qualitative variables were analyzed using the χ^2^ test or Fisher exact test. Continuous variables were compared using the *t* test or Mann-Whitney *U* test for nonnormal distribution variables. Survival curves were depicted using the Kaplan-Meier method and compared using the log-rank test. Independent prognostic factors were identified through stepwise selection in a Cox regression model. Added variables that were significantly related to survival in the univariate models (*P* < .10) were subsequently included in the multivariate Cox model. Cox proportional hazard ratios (HRs) were calculated to test correlation between clinical parameters and OS. Multivariate logistic regression analysis was performed to determine the association between clinical parameters and SRD of 6 months or more. Patient information was collected from January 1, 2018, to March 31, 2018, and analysis of these data was performed in April 2018. Statistical analyses to identify prognostic factors were performed using SPSS statistical software for Windows, version 23.0 (IBM). Two-sided *P* < .05 was considered statistically significant.

### 

## Results

### Patient Characteristics

A total of 2734 total patients (2499 of 2734 [91.4%] male; median [range] age, 56.5 [18-75] years) were included in the analysis. During follow-up, 180 patients received curative therapies (123 received ablation and 57 received resection) in the primary cohort. The corresponding figure in the validation cohort was 63 (38 received ablation and 25 received resection). These patients were excluded from the SRD analysis. In total, 2403 patients were included in the SRD analysis in the primary cohort and 331 in the validation cohort (Figure 1; eFigure 1 in the Supplement). The baseline characteristics of the patients in the primary and validation cohorts are shown in Table 1. In total, 32 patients were lost to follow-up in the study. Sixty-five patients with missing values of radiological images were excluded. In the study, the median (range) tumor size was 5.5 (2.0-16.5) cm; 40.1% of patients had tumors larger than 10 cm and 27.3% of patients had tumor extent more than 50%.

**Table 1.** Demographic and Clinicopathologic Characteristics of Primary Cohort for Sustained Response Duration Analysis

Characteristic	Primary Cohort (n = 2403)	Validation Cohort (n = 331)
Age, median (range), y	57 (18-75)	56.2 (18-75)
Sex, male/female, No.	2190/213	309/22
Hepatitis B virus, yes/no, No.	2000/403	290/41
Hepatitis C virus, yes/no, No.	49/2354	6/325
Nonalcoholic fatty liver disease, yes/no, No.	252/2151	37/294
Alcoholic liver disease, yes/no, No.	134/2269	21/310
α-Fetoprotein, median (range), ng/mL	803 (0-138 400)	822 (0-138 400)
Carbohydrate antigen 19-9, median (range), U/ml	10.0 (0.5-40.0)	15.0 (0.5-40.0)
γ-Glutamyltransferase, median (range), U/L	192.0 (10.0-950.0)	181.0 (10.0-950.0)
Alkaline phosphatase, median (range), U/L	70.0 (36.0-856.0)	87.5 (40.0-850.0)
Aspartate aminotransferase, median (range), U/L	42.0 (8.0-260.0)	40.0 (8.0-210.0)
Alanine transaminase, median (range), U/L	45.0 (10.0-314.0)	42.0 (10.0-324.0)
Albumin, median (range), g/L	36.0 (29.0-47.0)	35.0 (29.0-45.0)
Total bilirubin, median (range), mg/dL	0.9 (0.4-1.8)	0.8 (0.4-1.9)
Prothrombin time, median (range), s	12.0 (10.0-16.0)	12.2 (10.4-16.5)
White blood cell count, median (range), per μL	5000 (4000-10 000)	5000 (4000-10 000)
Hemoglobin, median (range), g/dL	12.2 (11.0-15.0)	12.0 (11.0-15.0)
Platelet count, median (range), ×10^3^/μL	110.0 (80.0-440.0)	110.0 (90.0-440.0)
Creatinine, median (range), mg/dL	1.2 (0.4-1.5)	1.2 (0.5-1.4)
Cirrhosis, yes/no, No.	1618/785	209/122
Child-Pugh classification, A/B, No.	2159/184	300/31
Ascites, yes/no, No.	98/2305	20/311
Indocyanine green retention at 15 min, ≤10%/>10%, No.	2303/380	303/28
Tumor size, median (range), cm	5.5 (2.0-16.5)	5.4 (2.0-16.0)
≤3/>3 Tumors, No.	508/1895	59/272
Bilobar disease, yes/no, No.	1768/635	258/73
Extent of disease within liver, ≤50%/>50%, No.	1530/573	260/71
Capsule, present/absent, No.	956/1447	125/206
Use of sorafenib, yes/no, No.	626/1677	91/240

SI conversions factors: To convert α-fetoprotein to μg/L, multiply by 1.0; γ-glutamyltransferase, alkaline phosphatase, aspartate aminotransferase, and alanine transaminase to μkat/L, multiply by 0.0167; total bilirubin to μmol/L, multiply by 17.104; white blood cell count to ×10^9^/L, multiply by 0.001; hemoglobin to g/L, multiply by 10.0; platelet count to ×10^9^/L, multiply by 1.0; and creatinine to μmol/L, multiply by 88.4.

### Radiological Response After cTACE

After the initial cTACE, CR was achieved in 382 of 2583 patients (14.8%), PR in 492 (19.0%), and SD in 1129 (43.7%). Progressive disease was observed in 580 patients (22.5%). The response outcomes and therapies after repeated cTACE are shown in Table 2 and Figure 1. According to radiological response, 201 patients achieved SRD of 12 months or more; 229, between 6 and 12 months; 656, between 3 and 6 months; and 837, less than 3 months. In the primary cohort, 430 patients achieved SRD of 6 months or more; 874, initial response; and 1032, best response. The median (range) ages of patients who achieved SRD of 6 months or more, initial responders, and best responders were 57 (18-75), 56 (18-75), and 57 (18-75) years, respectively. The percentages of male patients in these 3 groups were 90% (387 of 430), 87% (760 of 874), and 91% (939 of 1032), respectively.

**Table 2.** Response Outcome After Repeated cTACE During Follow-Up

Response	Indication to Repeated cTACE				
	Recurrence After CR, No. (%)	Non-CR			
		PR	SD	PD	Patients Experiencing Outcome, %
Tumor response to second cTACE					
CR	86 (57.3)	76	14	83	11.0
PR	25 (16.7)	78	110	112	19.0
SD	10 (6.7)	5	660	201	54.8
PD	29 (19.3)	2	59	179	15.2
Tumor response to third cTACE					
CR	61 (48.8)	55	6	29	13.3
PR	53 (42.4)	43	61	17	17.9
SD	1 (0.8)	1	379	2	56.6
PD	10 (8)	1	57	24	12.2
Tumor response to fourth cTACE					
CR	21 (39.6)	32	7	12	17.3
PR	24 (45.3)	38	48	5	30.8
SD	2 (3.8)	2	120	1	41.7
PD	6 (11.3)	1	20	9	10.2

Abbreviations: CR, complete response; cTACE, conventional transarterial chemoembolization; PD, progressive disease; PR, partial response; SD, stable disease.

### Optimal Predictable SRD Length Category for OS

By using the time-dependent area under the receiver operating characteristic curve (AUROC), SRD of 6 months or more was found to have the strongest association with 5-year OS after cTACE among the different durations of response (eTable 1 in the Supplement). The AUROC for SRD to determine 5-year OS was 0.913 (95% CI, 0.888-0.938) (eFigure 2A in the Supplement). Furthermore, the association between SRD and 5-year OS after cTACE was significantly stronger than that of the initial responder (AUROC, 0.840; 95% CI, 0.822-0.857; *P* < .001) (eFigure 2B in the Supplement) or the best responder (AUROC, 0.822; 95% CI, 0.807-0.838; *P* < .001) (eFigure 2C in the Supplement). Therefore, we chose SRD of 6 months or more as a potential prognostic factor of OS for further analysis.

### Overall Survival

**Figure 2.**
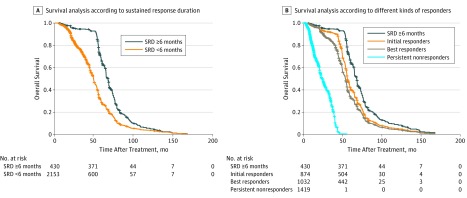
Survival Analysis According to Tumor Response A, Survival according sustained response duration (SRD) of less than 6 months vs 6 months or more. B, Survival among patients with SRD of 6 months or more, initial responders, best responders, and persistent nonresponders.

For patients with SRD of 6 months or more, the median (range) OS was 67.7 (64.8-72.1) months, which was better than that of patients with SRD of less than 6 months (median [range] OS, 53.5 [52.5-55.4] months) (HR, 0.132; 95% CI, 0.112-0.168; *P* < .001) (Figure 2A).

For patients with SRD of 6 months or more, initial responders, best responders, and persistent nonresponders, median (range) OS was 67.7 (64.8-72.1), 55.8 (55.0-57.7), 53.2 (52.2-54.6), and 23.6 (21.9-27.5) months, respectively. Patients with SRD of 6 months or more had better OS than initial responders (HR, 1.723; 95% CI, 1.620-1.832; *P* < .001) and best responders (HR, 1.870; 95% CI, 1.775-1.971; *P* < .001) (Figure 2B).

### Subgroup Analysis of OS

For tumor size of 5 cm or less, patients with SRD of 6 months or more had better OS than initial responders (77.0 vs 62.7 months; difference, 14.3 months; 95% CI, 13.3-14.9 months; *P* < .001) and best responders (77.0 vs 59.4 months; difference, 17.6 months; 95% CI, 16.8-18.2 months; *P* < .001). For tumor size larger than 5 cm, patients with SRD of 6 months or more had better OS than initial responders (64.8 months vs 53.2 months; difference, 11.6 months; 95% CI, 11.1-12.5 months; *P* < .001) and best responders (64.8 months vs 51.5 months; difference, 13.3 months; 95% CI, 12.4-14.1 months; *P* < .001) (eTable 2 in the Supplement).

For patients with 3 or fewer tumors, those with SRD of 6 months or more had better OS than initial responders (74.6 months vs 64.3 months; difference, 10.3 months; 95% CI, 9.6-11.5 months; *P* < .001) and best responders (74.6 months vs 61.1 months; difference, 13.5 months; 95% CI, 13.0-14.3 months; *P* < .001). For patients with more than 3 tumors, those with SRD of 6 months or more had better OS than initial responders (66.2 months vs 54.1 months; difference, 12.1 months; 95% CI, 11.5-12.4 months; *P* < .001) and best responders (66.2 months vs 52.5 months; difference, 13.7 months; 95% CI, 13.3-14.9 months; *P* < .001) (eTable 2 in the Supplement).

For tumors with a capsule, patients with SRD of 6 months or more had better OS than initial responders (78.4 months vs 65.4 months; difference, 13.0 months; 95% CI, 12.6-13.7 months; *P* < .001) and best responders (78.4 months vs 62.3 months; difference, 16.1 months; 95% CI, 15.4-16.7 months; *P* < .001). For tumors with no capsule, patients with SRD of 6 months or more had better OS than initial responders (62.5 months vs 51.2 months; difference, 11.3 months; 95% CI, 10.7-12.1 months; *P* < .001) and best responders (62.5 months vs 49.5 months; difference, 13.0 months; 95% CI, 12.1-13.8 months; *P* < .001) (eTable 2 in the Supplement).

### Prognostic Significance of SRD

Multivariate analysis was performed to evaluate the prognostic significance of SRD. Considering there was close association between the initial and the best response (eTable 3 in the Supplement), they were separately entered into 2 multivariate analyses with SRD. In multivariate analysis A, which included initial response, SRD of 6 months or more (HR, 0.145; 95% CI, 0.124-0.170; *P* < .001) and the initial response (HR, 0.406; 95% CI, 0.358-0.460; *P* < .001) were found to be the independent prognostic factors for OS. In multivariate analysis B, which included best response, SRD of 6 months or more (HR, 0.142; 95% CI, 0.121-0.174; *P* < .001) and the best response (HR, 0.769; 95% CI, 0.673-0.878; *P* < .001) were found to be the independent prognostic factors for OS (Table 3). According to the results of multivariate analysis, the risk of death for patients with SRD of 6 months or more was reduced 85%, more than that of those of initial responders (almost 60%) and best responders (almost 30%).

**Table 3.** Prognostic Significance of Sustained Response Duration for Overall Survival

Variables	Univariate Analysis		Multivariate Analysis A		Multivariate Analysis B	
	HR (95% CI)	*P* Value	HR (95% CI)	*P* Value	HR (95% CI)	*P* Value
Age (≤60 vs >60 y)	0.910 (0.785-1.055)	.43				
Gender (male vs female)	1.204 (0.953-1.521)	.13				
Hepatitis B virus (yes vs no)	1.010 (0.907-1.125)	.78				
Hepatitis C virus (yes vs no)	0.931 (0.729-1.190)	.65				
α-Fetoprotein (≤400 vs >400 ng/mL)	0.844 (0.652-1.093)	.18				
Carbohydrate antigen 19-9 (≤37 vs >37 U/ml)	1.054 (0.965-1.151)	.22				
γ-Glutamyltransferase (≤50 vs >50 U/L)	1.070 (0.994-1.151)	.06				
Alkaline phosphatase (≤110 vs >110 U/L)	1.041 (0.930-1.167)	.44				
Aspartate aminotransferase (≤40 vs >40 U/L)	1.441 (1.288-1.612)	<.001	1.357 (1.209-1.522)	<.001	1.342 (1.196-1.506)	<.001
Alanine transaminase (≤40 vs >40 U/L)	1.181 (0.963-1.448)	.11				
Albumin (≤35 vs >35 g/L)	0.860 (0.731-1.013)	.07				
Total bilirubin (≤1.2 vs >1.2 mg/dL)	1.121 (0.930-1.351)	.10				
Prothrombin time (≤14 vs >14 s)	0.955 (0.788-1.158)	.65				
White blood cell count (≤4000/μL vs >4000/μL)	1.009 (0.896-1.136)	.82				
Hemoglobin (≤12 vs >12 g/dL)	1.305 (0.886-1.209)	.79				
Platelet count (≤100 vs >100 × 10^3^/μL)	0.927 (0.835-1.030)	.36				
Creatinine (≤1.0 vs >1.2 mg/dL)	1.042 (0.946-1.148)	.12				
Cirrhosis (yes vs no)	0.893 (0.744-1.073)	.07				
Child-Pugh classification (A vs B)	1.103 (0.965-1.272)	.12				
Ascites (yes vs no)	0.919 (0.804-1.050)	.27				
Indocyanine green retention at 15 min (≤10% vs >10%)	1.074 (0.991-1.164)	.06				
Tumor size (≤5 vs >5 cm)	2.169 (1.651-2.851)	<.001	1.802 (1.486-2.185)	<.001	1.731 (1.515-1.977)	<.001
Tumor No. (≤3 vs >3)	2.263 (2.041-2.509)	<.001	1.840 (1.609-2.103)	<.001	1.774 (1.464-2.105)	<.001
Bilobar disease (yes vs no)	0.993 (0.897-1.100)	.89				
Extent of disease within liver (≤50% vs >50%)	0.979 (0.879-1.091)	.97				
Capsule (absent vs present)	0.532 (0.478-0.593)	<.001	0.723 (0.620-0.843)	<.001	0.703 (0.626-0.851)	<.001
Use of sorafenib (yes vs no)	0.954 (0.855-1.064)	.40				
Objective response as the initial response^a^	0.267 (0.236-0.303)	<.001	0.406 (0.358-0.460)	<.001		
Objective response as the best response^a^	0.582 (0.526-0.645)	<.001			0.769 (0.673-0.878)	<.001
Sustained response duration (<6 vs ≥6 mo)	0.132 (0.112-0.168)	<.001	0.145 (0.124-0.170)	<.001	0.142 (0.121-0.174)	<.001

Abbreviation: HR, hazard ratio.

SI conversion factors: To convert α-fetoprotein to μg/L, multiply by 1.0; γ-glutamyltransferase, alkaline phosphatase, aspartate aminotransferase, and alanine transaminase to μkat/L, multiply by 0.0167; total bilirubin to μmol/L, multiply by 17.104; white blood cell count to ×10^9^/L, multiply by 0.001; hemoglobin to g/L, multiply by 10.0; platelet count to ×10^9^/L, multiply by 1.0; and creatinine to μmol/L, multiply by 88.4.

^a^ Objective response was defined as the sum of complete response and partial response. Considering there was close correlation between the initial response and the best response, they were separately entered into 2 multivariate analyses with sustained response duration. Initial response was included in multivariate analysis A, and best response was included in multivariate analysis B.

### Clinical Factors Associated With SRD of 6 Months or More

Using multivariate analysis, we found that tumor number (≤3 vs >3: odds ratio [OR], 3.177; 95% CI, 1.458-6.923; *P* = .004), tumor size (≤5 vs >5 cm: OR, 1.687; 95% CI, 1.256-2.266; *P* = .001), and capsule (absent vs present: OR, 0.478; 95% CI, 0.369-0.619; *P* < .001) were significantly associated with SRD of 6 months or more (eTable 4 in the Supplement).

### Significance of SRD in the Validation Cohort

The response outcomes and therapies after repeated cTACE in the validation cohort are shown in eTable 5 and eFigure 1 in the Supplement. The numbers of patients who achieved SRD of 12 months or more, between 6 and 12 months, between 3 and 6 months, and less than 3 months were 37, 30, 45, and 219, respectively.

For patients with SRD of 6 months or more, initial responders, best responders, and persistent nonresponders, median OS was 48.0, 34.4, 23.6, and 13.6 months, respectively. Patients with SRD of 6 months or more had better OS than initial responders (HR, 1.969; 95% CI, 1.712-2.264; *P* < .001) and best responders (HR, 2.407; 95% CI, 1.902-3.045; *P* < .001) (eFigure 3 in the Supplement).

The AUROC for SRD of 6 months or more to determine 5-year OS was 0.929 (95% CI, 0.898-0.960), which was better than that of initial response (0.865; 95% CI, 0.840-0.890; *P* < .001) and best response (0.822; 95% CI, 0.781-0.863; *P* < .001) (eFigure 4 in the Supplement).

## Discussion

Although OS is the primary end point in clinical studies related to HCC, objective response based on mRECIST has been reported to be a reliable second end point.^19^ However, this study enrolled patients with advanced HCC, and the therapy was the target drug. It may be not appropriate to use objective response as a surrogate end point in intermediate HCC owing to different tumor stages and different treatments. Furthermore, continuing administration of target drugs is the best choice for patients with advanced HCC who achieve objective response. However, as the main treatment for intermediate HCC, cTACE needs to be performed repeatedly. A study is needed to evaluate the role of objective response in intermediate HCC after cTACE and also to find other candidates more reliable than objective response as secondary end point.

Interestingly and critically, our large cohort study demonstrated that SRD of 6 months or more was more strongly associated with OS than initial responder or best responder status. Patients with SRD of 6 months or more had longer survival time than did initial or best responders. Multivariate analysis showed that the death risk of patients with SRD of 6 months or more was reduced 85%, more than that of initial responders (60%) and best responders (30%). Subgroup analysis further showed the OS benefit persisted for patients with SRD of 6 months or more regardless of tumor size, tumor number, or capsule status. These data demonstrate that among the patients with objective response, such as CR, some factors other than radiological response, such as tumor biology and tumor microenvironment, may alter the efficacy of cTACE and result in a different long-term survival.^20,21,22^ Thus, we propose that SRD is a good candidate to reflect the net result of the interactions between tumor cells, liver disease biology, and tumor microenvironment, and can be used in clinical practice as an excellent and reliable predictor of outcomes. The use of SRD as a surrogate end point in clinical trials would allow new strategies to be discarded early and enable testing new therapies. Furthermore, we validated our results on clinical significance of SRD in another cohort of 331 patients with HCC treated at the First Affiliated Hospital, Sun Yet-sen University. This result strongly suggested that the patients with SRD of 6 months or more can be more appropriately defined as responders after cTACE. After regression analysis, we found that tumor number, tumor size, and tumor capsule were significantly associated with SRD of 6 months or more. It is reasonable to conclude that tumor burden (tumor number, tumor size), and tumor structure (tumor capsule) play important roles in determining overall success rates of cTACE procedures.^14,23,24,25^

It is of great importance to know what the treatment strategy is for patients to achieve objective response or just SD during clinical practice. Repetition of cTACE has been proven to increase the tumor response rate, thereby prolonging survival. However, repeated cTACE can cause adverse effects, such as liver damage or gastrointestinal bleeding, which results in morbidity. Two strategies of repeated cTACE have been advocated recently: performing cTACE courses at regular intervals (on schedule), or performing cTACE on demand. The latter strategy seems to be the most rational and effective procedure because it is inherently more individualized and tailored for tumor response and tolerance of procedure. It has also been speculated to be able to reduce the degree of liver damage and complications. However, there are no definitive data demonstrating which treatment strategy is superior. Indirectly, our results lend support to the on demand strategy. For example, in a patient with sustained CR of more than 6 months, there may be no need for further courses of cTACE. Even for patients who did not achieve CR, maintaining the status of PR or SD may be a reasonable scenario during follow-up with minimal courses of cTACE, especially for patients who tolerate cTACE or other treatments poorly. Furthermore, the findings of the study may be useful for patients on the waiting list for liver transplantation. For patients who achieved sustained response and had tumor characteristics that met the criteria of liver transplantation, the longer time of SRD (≥6 months) may reflect the fact that lesions after cTACE are less prone to local or systemic progression, and this allows patients to remain on the waiting list until an organ becomes available.

### Limitations

We acknowledge that our study has several limitations. First, the initial CR and PR and the best response of CR and PR were lower in our study when compared with other studies.^9,26,27^ Lower response rates were most likely due to the fact that the tumor burden was greater in our study, with a median (range) tumor size of 5.5 (2.0-16.5) cm, 40.1% of patients having tumors larger than 10 cm, and 27.3% of patients with tumor extent more than 50%. Additionally, our study was conducted in a cohort of largely monoethnic Chinese patients with different demographics and underlying causes of liver disease, which may have contributed to the lower response rate. The heterogeneity of the treatment protocol may be another contributor to lower response rate. Second, a large part of our cohort was analyzed retrospectively, which creates inherent limitations, including potential selection, measurement, and misclassification biases. Third, a mixture of either CT or MRI was used to define response after cTACE. Some investigators consider MRI superior to CT for detection of viable tumor residuals after lipiodol-based cTACE, and some think MRI should be used as the first choice for response assessment after cTACE.^28^ Finally, the cTACE procedure used in our study is not universally used in other countries, which will require validation in other centers and regions. Clearly, more prospective studies in other areas are needed to further validate the association between SRD and OS before our findings can be generalized.

## Conclusions

In this study, analysis of a large cohort of Chinese patients demonstrated that SRD of 6 months or more after cTACE is the most significant prognostic factor in patients with intermediate HCC.

## Notes

Supplement. **eTable 1.** AUROC for Different Time Point of Sustained Response Duration **eTable 2.** Subgroup Analysis for Overall Survival in Primary Cohort **eTable 3.** The Initial and Best Response in Detail **eTable 4.** Clinical Factors Associated With Sustain Response Duration ≥6 Months After the First TACE **eTable 5.** Response Outcome After Repeated cTACE During Follow-up **eFigure 1. **Flow Chart Summarizing the Actual Conventional cTACE Repetition in the Validation Cohort Based on a Schedule “On Demand” **eFigure 2.** Time-Dependent Area Under the Receiver-Operating Characteristic Curve (AUROC) of the Initial Response (A), the Best Response (B) and SRD (C) in Primary Cohort **eFigure 3.** Kaplan-Meier Curves Generated Among Validation Cohort to Compare Survival Among Patients With Sustained Response Duration ≥6 Months, Initial Responders, Subsequent Responders, Persistent Non-responders **eFigure 4.** Time-Dependent Area Under the Receiver-Operating Characteristic Curve (AUROC) of the Initial Response (A), the Best Response (B) and SRD (C) in Validation Cohort
