# Supplementary material for: Association of Sustained Response Duration With Survival After Conventional Transarterial Chemoembolization in Patients With Hepatocellular Carcinoma
Source: JAMA Netw Open. 2018 Oct 5;1(6):e183213. doi: 10.1001/jamanetworkopen.2018.3213 (PMC6324454; doi:10.1001/jamanetworkopen.2018.3213)
Supplement: Supplement. — eTable 1. AUROC for Different Time Point of Sustained Response Duration eTable 2. Subgroup Analysis for Overall Survival in Primary Cohort eTable 3. The Initial and Best Response in Detail eTable 4. Clinical Factors Associated With Sustain Response Duration ≥6 Months After the First TACE eTable 5. Response Outcome After Repeated cTACE During Follow-up eFigure 1. Flow Chart Summarizing the Actual Conventional cTACE Repetition in the Validation Cohort Based on a Schedule “On Demand” eFigure 2. Time-Dependent Area Under the Receiver-Operating Characteristic Curve (AUROC) of the Initial Response (A), the Best Response (B) and SRD (C) in Primary Cohort eFigure 3. Kaplan-Meier Curves Generated Among Validation Cohort to Compare Survival Among Patients With Sustained Response Duration ≥6 Months, Initial Responders, Subsequent Responders, Persistent Non-responders eFigure 4. Time-Dependent Area Under the Receiver-Operating Characteristic Curve (AUROC) of the Initial Response (A), the Best Response (B) and SRD (C) in Validation Cohort [file jamanetwopen-1-e183213-s001.pdf]

# Supplementary Online Content

Zhang Y, Zhang M, Chen M, et al. Association of Sustained Response Duration With Survival After Conventional Transarterial Chemoembolization in Patients With Hepatocellular Carcinoma. *JAMA Netw Open.* 2018;1(6):e183213. doi:10.1001/jamanetworkopen.2018.3213

eTable 1. AUROC for Different Time Point of Sustained Response Duration

eTable 2. Subgroup Analysis for Overall Survival in Primary Cohort

eTable 3. The Initial and Best Response in Detail

eTable 4. Clinical Factors Associated With Sustain Response Duration ≥6 Months After the First TACE

eTable 5. Response Outcome After Repeated cTACE During Follow-up

eFigure 1. Flow Chart Summarizing the Actual Conventional cTACE Repetition in the Validation Cohort Based on a Schedule “On Demand”

eFigure 2. Time-Dependent Area Under the Receiver-Operating Characteristic Curve (AUROC) of the Initial Response (A), the Best Response (B) and SRD (C) in Primary Cohort

eFigure 3. Kaplan-Meier Curves Generated Among Validation Cohort to Compare Survival Among Patients With Sustained Response Duration ≥6 Months, Initial Responders, Subsequent Responders, Persistent Non-responders

eFigure 4. Time-Dependent Area Under the Receiver-Operating Characteristic Curve (AUROC) of the Initial Response (A), the Best Response (B) and SRD (C) in Validation Cohort

© 2018 Zhang Y et al. *JAMA Network Open.*

This supplementary material has been provided by the authors to give readers additional information about their work.

© 2018 Zhang Y et al. *JAMA Network Open.*

**eTable 1.** AUROC for different time point of sustained response duration

| | SRD | | | | |
|---|---|---|---|---|---|
| | ≥1 month | ≥3 months | ≥6 months | ≥9 months | ≥12 months |
| AUROC | 0.665 (95% CI 0.640-0.690) | 0.785 (95% CI 0.743-0.837) | 0.913 (95% CI 0.888-0.938) | 0.856 (95% CI 0.826-0.886) | 0.844 (95% CI 0.803-0.885) |

AUROC: time-dependent area under the receiver-operating characteristic curve;

SRD: sustained response duration.

© 2018 Zhang Y et al. *JAMA Network Open.*

**eTable 2.** Subgroup analysis for overall survival in primary cohort

| | Median overall survival | | | |
|---|---|---|---|---|
| | SRD ≥6m | Initial responder | Best responder | *p* value |
| Tumor size ≤5cm | 77.0 | 62.7 | 59.4 | <0.001 |
| Tumor size >5cm | 64.8 | 53.2 | 51.5 | <0.001 |
| Tumor number ≤3 | 74.6 | 64.3 | 61.1 | <0.001 |
| Tumor number >3 | 66.2 | 54.1 | 52.5 | <0.001 |
| Capsule | 78.4 | 65.4 | 61.2 | <0.001 |
| Non-capsule | 62.5 | 51.2 | 49.5 | <0.001 |

SRD sustained response duration.

© 2018 Zhang Y et al. *JAMA Network Open.*

**eTable 3. The initial and best response in detail.** After initial cTACE, CR was achieved in 382 of 2538 (14.8%) patients, PR in 492 (19.0%), and SD in 1129 (43.7%). PD was observed in 580 (22.5%) patients. After four times of cTACE, overall, the best response consisted of CR in 742 (28.7%) patients, PR in 300 (11.6%), and SD in 684 (26.5%). Thus the number of patients with objective response (CR+PR) increased from 874 (33.8%) to 1042 (40.3%) after repeated TACE sessions.

| | | **Best response** | | | |
|---|---|---|---|---|---|
| | | CR | PR | SD | PD |
| **Initial response** | CR | 382 | 0 | 0 | 0 |
| | PR | 174 | 78 | 0 | 0 |
| | SD | 103 | 110 | 470 | 0 |
| | PD | 83 | 112 | 201 | 184 |

© 2018 Zhang Y et al. *JAMA Network Open.*

**eTable 4.** Clinical factors associated with sustain response duration ≥6 months after the first TACE

| Variables | Univariate analysis | Multivariate analysis | |
|---|---|---|---|
| | *p* value | *p* value | OR (95% CI) |
| Age(≤60/>60y) | 0.18 | | |
| Gender(Male/Female) | 0.16 | | |
| HBV(yes/no) | 0.40 | | |
| HCV(yes/no) | 0.15 | | |
| AFP(≤400/>400ng/ml) | 0.16 | | |
| CA199(≤37/>37U/ml) | 0.13 | | |
| GGT(≤37/>37 u/L), | 0.17 | | |
| ALP(≤110/>110 U/L) | 0.17 | | |
| AST(≤40/>40 u/L) | <0.001 | | |
| ALT(≤40/>40 u/L) | 0.17 | | |
| ALB(≤35/>35 g/L) | 0.32 | | |
| TBIL(≤20.5/>20.5 umol/L) | 0.23 | | |
| PT(≤14/>14 s) | 0.18 | | |
| WBC(≤4/>4*10E$^9$/L) | 0.16 | | |
| HB(≤120/>120 g/L) | 0.33 | | |
| PLT(≤100/>100*10E$^9$/L) | 0.94 | | |
| Cr(≤110/>110 umol/L) | 0.17 | | |
| Cirrhosis(yes/no) | 0.32 | | |
| Child-Pugh classification(A/B) | 0.23 | | |
| Ascites(yes/no) | 0.10 | | |
| ICGR15 (≤10% />10%) | 0.03 | | |
| Tumor size (≤5/>5cm) | <0.001 | 0.001 | 1.687 (1.256-2.266) |
| Tumor number (1/>1) | <0.001 | 0.004 | 3.177 (1.458-6.923) |
| Bilobar disease (yes/no) | 0.79 | | |
| Extent (≤50%/>50%) | 0.12 | | |

© 2018 Zhang Y et al. *JAMA Network Open.*

| | | | |
|---|---|---|---|
| Capsule (yes/no) | <0.001 | <0.001 | 0.478 (0.369-0.619) |

HBV, hepatitis B virus; HBV, hepatitis C virus; AFP, alpha fetoprotein; CA199, carbohydrate antigen 19-9; GGT, gamma-glutamyl transpeptidase; ALP, Alkaline phosphatase; AST, aspartate aminotransferase; ALT, alanine transaminase; ALB, albumin; PT, prothrombin time; TBIL, total bilirubin; WBC, white blood cell; Hb, hemoglobin; PLT, platelet; Cr, creatinine; ICGR15, indocyanine green retention at 15 min.

© 2018 Zhang Y et al. *JAMA Network Open.*

**eTable 5.** Response outcome after repeated cTACE during follow-up

| | | Indication to repeat cTACE | | | | | |
|---|---|---|---|---|---|---|---|
| | | Recurrence after CR | | Non-CR | | | |
| | | n | % | PR | SD | PD | % |
| Tumor response to 2nd TACE | CR | 15 | 53.6 | 14 | 5 | 3 | 9.1 |
| | PR | 6 | 21.4 | 10 | 21 | 16 | 19.5 |
| | SD | 2 | 7.1 | 0 | 102 | 34 | 56.4 |
| | PD | 5 | 17.9 | 1 | 9 | 26 | 19.0 |
| Tumor response to 3rd TACE | CR | 13 | 52.0 | 9 | 1 | 6 | 14.4 |
| | PR | 9 | 36.0 | 12 | 10 | 5 | 24.3 |
| | SD | 1 | 4.0 | 0 | 56 | 0 | 50.5 |
| | PD | 3 | 12.0 | 1 | 8 | 3 | 10.8 |
| Tumor response to 4th TACE | CR | 5 | 38.4 | 7 | 1 | 2 | 15.4 |
| | PR | 6 | 46.2 | 12 | 8 | 1 | 32.3 |
| | SD | 0 | 0 | 0 | 26 | 0 | 40.0 |
| | PD | 2 | 15.4 | 1 | 5 | 2 | 12.3 |

cTACE, conventional transarterial chemoembolization; CR: complete response; PR: partial response; SD: stable disease; PD: progressive disease.

© 2018 Zhang Y et al. *JAMA Network Open.*

**eFigure 1**：Flow chart summarizing the actual conventional cTACE repetition in the validation cohort based on a schedule "on demand".

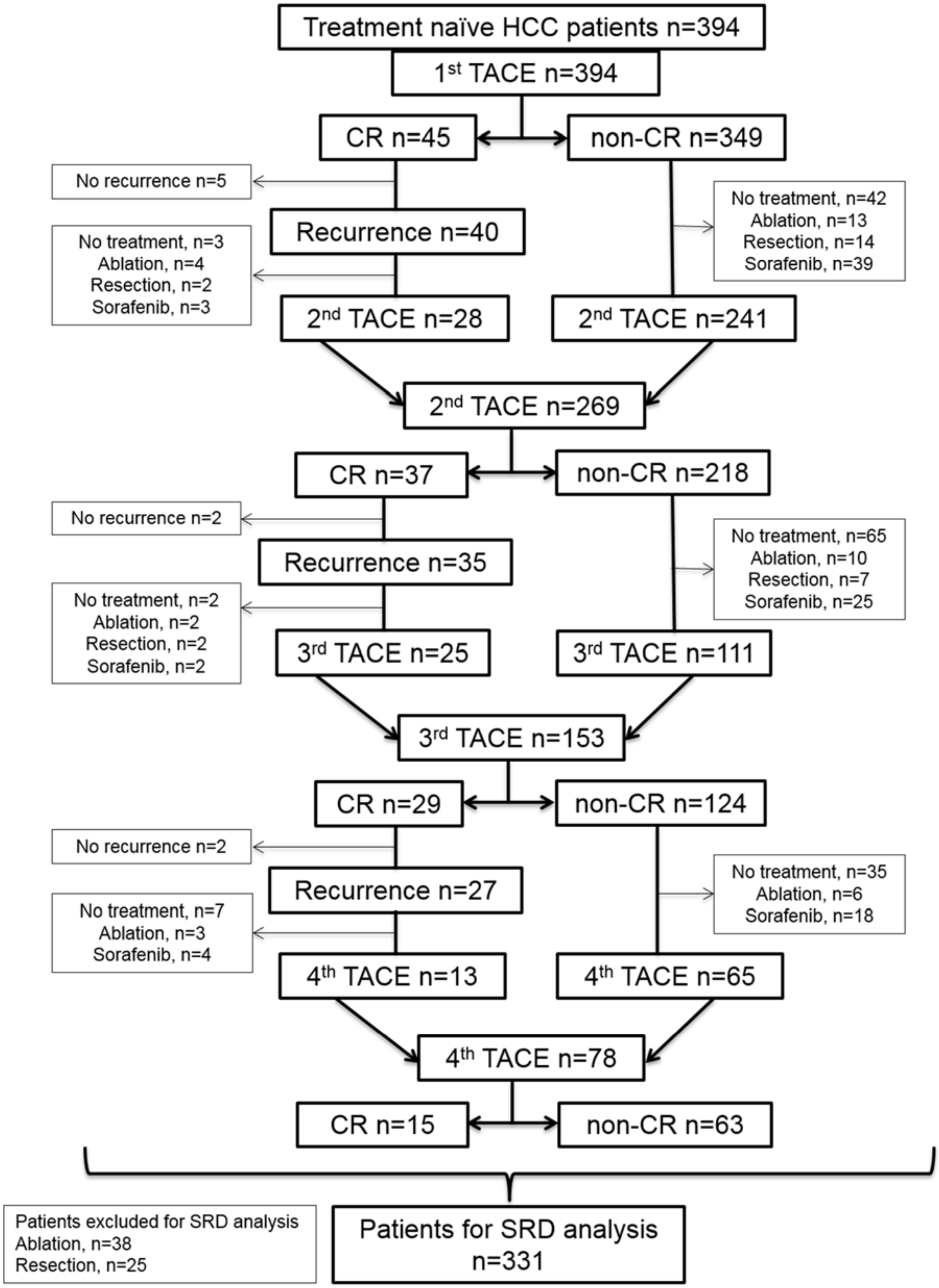


© 2018 Zhang Y et al. *JAMA Network Open.*

cTACE, conventional transarterial chemoembolization; CR, complete response; PR, partial response; SD, stable disease; PD, progressive disease; SRD, sustained response duration.

© 2018 Zhang Y et al. *JAMA Network Open.*

**eFigure 2:** Time-dependent area under the receiver-operating characteristic curve (AUROC) of the initial response (A), the best response (B) and SRD (C) in primary cohort.

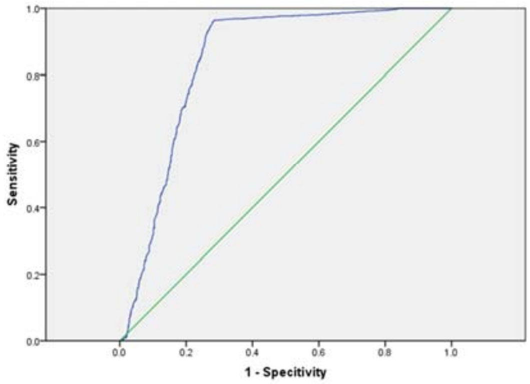


(A)

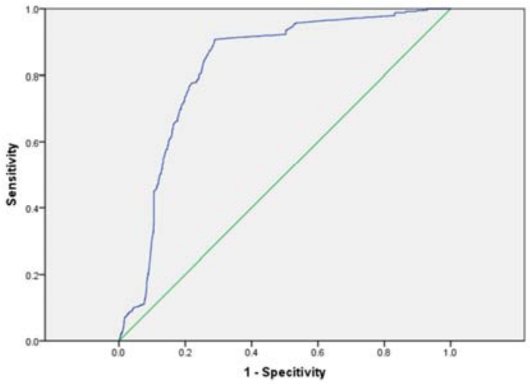


(B)

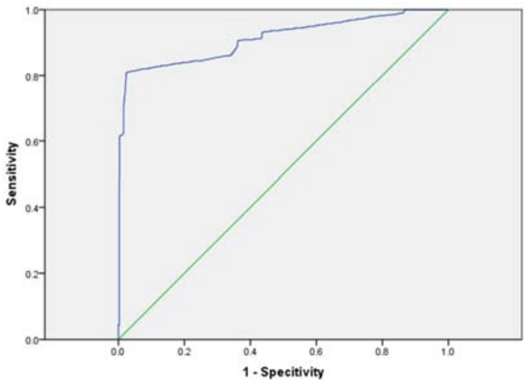


(C)

SRD, sustained response duration**.**

© 2018 Zhang Y et al. *JAMA Network Open.*

**eFigure 3:** Kaplan-Meier curves generated among validation cohort to compare survival among patients with sustained response duration ≥6 months, initial responders, subsequent responders, persistent non-responders.

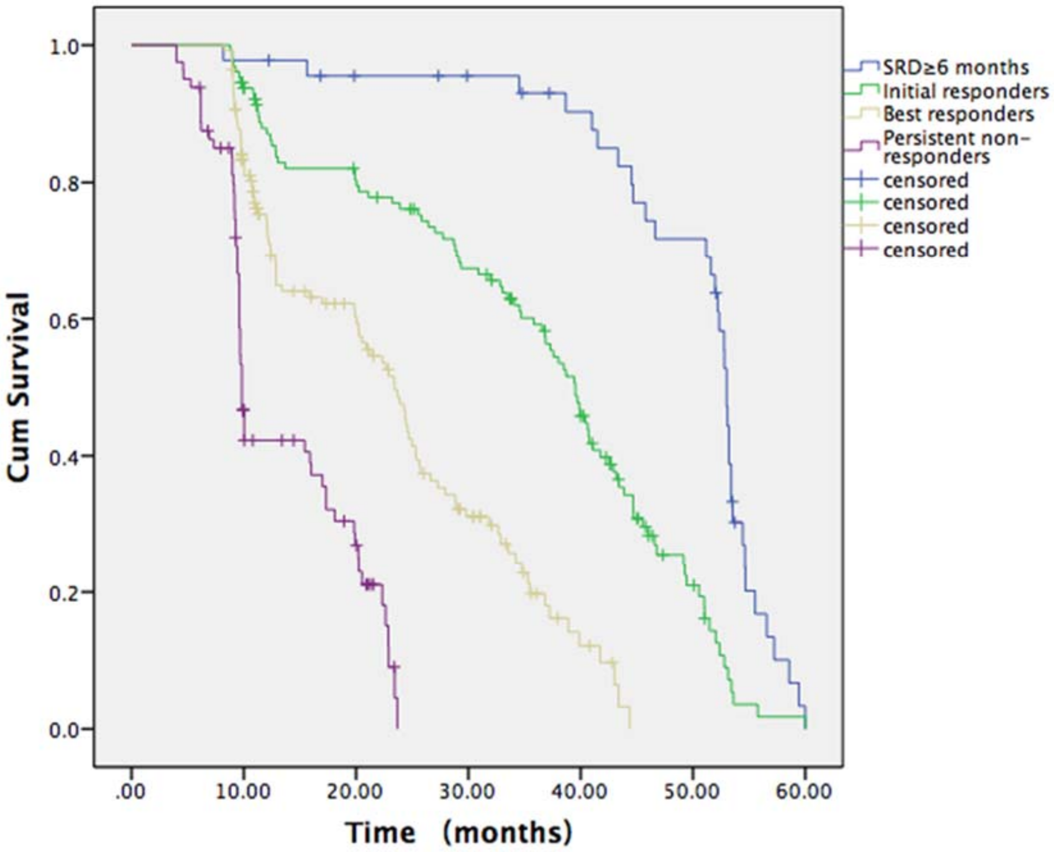


SRD, sustained response duration.

© 2018 Zhang Y et al. *JAMA Network Open.*

**eFigure 4:** Time-dependent area under the receiver-operating characteristic curve (AUROC) of the initial response (A), the best response (B) and SRD (C) in validation cohort.

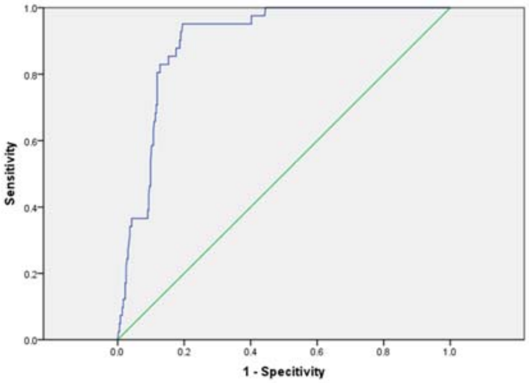


(A)

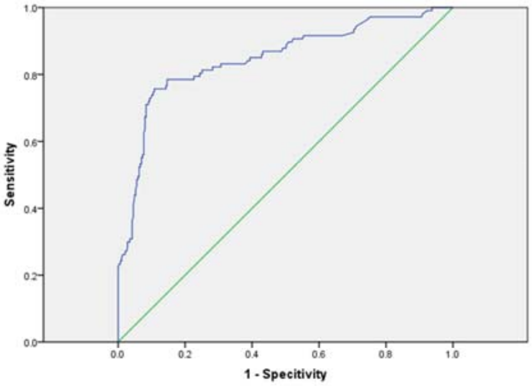


(B)

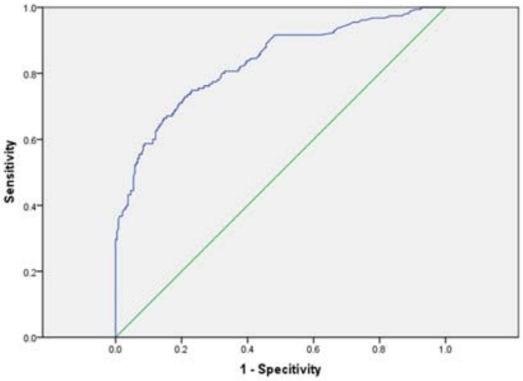


(C)

SRD, sustained response duration.

© 2018 Zhang Y et al. *JAMA Network Open.*
